# Retinol-binding protein 4 (RBP4) and high sensitivity C-reactive protein (hs-CRP) levels in patients with diminished ovarian reserve (DOR): a cross-sectional study

**DOI:** 10.1186/s12958-020-00670-4

**Published:** 2020-11-16

**Authors:** Wen Zou, Zehao Wang, Jing Xia, Jing Yang

**Affiliations:** 1 Reproductive Medical Center, Renmin Hospital of Wuhan University, Wuhan, 430060 China; 2 Hubei Clinic Research Center for Assisted Reproductive Technology and Embryonic Development, Wuhan, 430060 China

**Keywords:** Diminished ovarian reserve, Antioxidative stress, Retinol-binding protein 4, Oxidative stress, High-sensitivity C-reactive protein

## Abstract

**Background:**

Antioxidant/oxidant imbalance has been reported to be related to diminished ovarian reserve (DOR). Vitamin A (retinol), a kind of antioxidant, plays a role in restoring ovarian oxidative damage, while C-reactive protein (CRP) is the classical marker of oxidative stress and has recently been identified as an independent variable that is associated with low anti-Mullerian hormone (AMH) levels in young women with DOR. Additionally, retinol binding protein 4 (RBP4) can be considered a substitute for retinol in healthy, nonobese women. The study aim was to determine the relationship between serum RBP4, high sensitivity C-reactive protein (hs-CRP) concentrations and ovarian reserve in nonobese DOR patients.

**Methods:**

This study included 24 DOR women and 48 normal ovarian reserve (NOR) women from the reproductive medical center of Renmin Hospital of Wuhan University. The serum RBP4 and high-sensitivity CRP (hs-CRP) levels were measured with ELISA kits.

**Results:**

RBP4 levels (20,648.36 ± 5475.16 ng/ml vs 23,986.48 ± 5995.64 ng/ml, *p* = 0.025) were decreased, and hs-CRP levels (695.08 ± 1090.19 ng/ml vs 364.32 ± 786.29 ng/ml, *p* = 0.012) were increased in the DOR group. Serum RBP4 was positively related to AMH (Pearson *r* = 0.518, *p* = 0.000), while hs-CRP was negatively correlated with AMH (Spearman *r* = − 0.345, *p* = 0.005). after adjustments were made for the covariables, multiple line regression analysis showed that positive association between RBP4 and AMH still existed (β = 0.450, *p* < 0.001).

**Conclusion:**

Decreased serum RBP4 levels and increased serum hs-CRP were observed in DOR patients in our study, and the strong correlation between RBP4 and AMH supports the notion that oxidative stress plays a role in DOR, and that appropriate levels of antioxidant vitamin A may be protective against ovarian reserve dysfunction.

## Background

Diminished ovarian reserve (DOR) often results in reduced fecundability, accompanied by reduced oocyte quantity and decreased oocytes quality. These phenomena also occur during normal ovarian aging [1, 2]. Due to the close relationship between DOR with poor ovarian response, DOR patients usually have a higher rate of in vitro fertilization (IVF) failure [3, 4]. These difficulties are frequently encountered by reproductive physicians and are a major challenge in the field of reproductive medicine. Decreased oocyte capacity is believed to be partly attributed to oxidative stress and long-term use of low-dose antioxidants containing vitamin A has been demonstrated to favor ovarian function by decreasing the accumulation of reactive oxygen species (ROS) [5, 6].

Vitamin A, also known as retinol, is an antioxidant. Its important role in repairing oxidative damage to the ovary in conjunction with other antioxidative vitamins has been extensively studied [6–9]. However, retinol and its metabolites are involved in normal physiological ovarian follicle development and maturation in different species [10]. Data on the potential relationship between retinol levels and ovarian reserve are limited. Serum retinol-binding protein 4 (RBP4), a known transport protein of retinol, binds to retinol at a ratio of 1:1 and delivers retinol to peripheral target tissues. Therefore, serum RBP4 levels can predict retinol concentrations in the blood to a certain degree [11].

Oxidative stress occurs when the antioxidation system is weakened, at which time, ROS levels increase to induce chronic inflammation and increased levels of inflammatory cytokines. Among them, C-reactive protein (CRP) has been reported to play a key role in this association [12]. Some studies have shown that higher CRP concentrations are related to lower levels of retinol in inflammatory diseases [13–15], but other studies have failed to find this connection [16]. These contradictory results may be due to differences in the races of the study groups [17], the populations included [16] and detection methods used [18]. Furthermore, CRP has recently, been identified as a marker of cardiovascular risk that is inversely correlated with anti-Mullerian hormone (AMH) levels in DOR patients [19, 20]. However, so far, there are few reports on the relationships between RBP4, CRP and DOR. Our study was designed to investigate serum RBP4 and CRP concentrations in DOR patients and to further examine the possible link between AMH and RBP4 or, CRP levels.

## Materials and methods

### Subjects

We conducted a cross-sectional study of infertile women between May 2019 and October 2019 in the Reproductive Medical Center of Renmin Hospital of Wuhan University with a total of 72 infertile women aged 21–40 years who met our eligibility criteria. The Clinical Research Ethics Committee of Renmin Hospital of Wuhan University provided ethical approval for study (WDRY2019-K007), and all patients provided informed consent.

Before to our hospital, some patients usually have some indicators been tested in other hospitals, we divided all subjects according to their serum AMH values because all infertile women who initially visit our reproductive medical center must undergo AMH testing in our hospital. According to Cohen J, et al. [21], women with AMH < 1.1 ng/ml were classified into the DOR group, and those with AMH ≥ 1.1 ng/ml were classified into the normal ovarian reserve (NOR) group. Of these women, 24 in the DOR and 48 were in the NOR group. The NOR group comprised infertile subjects whose infertility was mainly attributed to unexplained infertility or male factors. Data on lifestyle, general health and factors affecting fecundability were acquired via questionnaires. The exclusion criteria were as follows: (1) presence or previous history of any metabolic, endocrine and infection disease; (2) obesity (body mass index (BMI), ≥28 kg/m^2^); (3) use of steroidal drugs or multivitamins containing vitamin A; (4) unilateral or bilateral ovarian surgery; (5) other factors related to fertility, such as smoking, alcohol consumption and diet; (6) family history of cardiovascular diseases or other metabolic diseases.

**Fig. 1:**
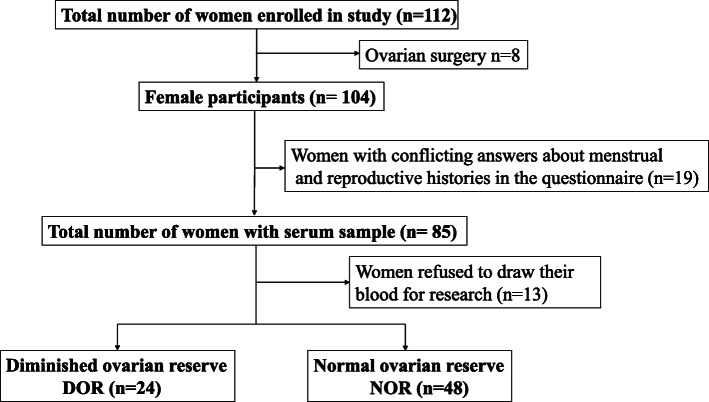
The flowchart of participants selection

Altogether, 112 infertile women were initially enrolled (Fig. 1). Of these, eight women had undergone ovarian-related surgeries. Nineteen women gave conflicting answers about their menstrual cycles and reproductive histories. Thirteen women refused to undergo a blood draw for research purposes. After exclusion of 40 subjects, 72 eligible subjects were finally enrolled in this study. Figure 1 illustrates the selection process of participants in a simplified flowchart.

### Clinical database data extraction

Data pertaining to the patients’ age, BMI, AMH levels, baseline E2 and day 3-FSH levels, day 2–5 total antral follicle count, years of infertility, numbers of oocytes retrieved and season of sample collection were obtained from the database.

### Blood sample collection

Fasting blood samples were collected regardless of the day of the menstrual cycle for the patient’s convenience. As soon as possible, the blood samples were transported in an ice box to the laboratory. Serum was obtained after centrifugation and then frozen at − 80 °C prior to the assay. Throughout all of the processing procedures, the samples were strictly protected from light and heat. In addition, female reproduction was reported to be affected by seasonal changes [22], we also recorded detailed information regarding their season of the blood sampling.

### Laboratory measurements of serum RBP4 and hs-CRP

Serum RBP4 and hs-CRP levels were measured using an enzyme-linked immunosorbent assay (ELISA) kit (Cusabio Biotech, Wuhan, China). The minimum detection limits and sensitivity values were 15.6 ng/mL and 3.9 ng/ml, respectively, for RBP4, and 0.625 ng/ml and 0.156 ng/ml, respectively, for hs-CRP. The intra-assay variation coefficients of RBP4 and hs-CRP are both less than 8%, and inter-assay variation coefficients of RBP4 and hs-CRP are both less than 10%.

### Statistics analysis

According to the normal distribution of variables, continuous variables are presented as means ± SDs or medians ± interquartile ranges (IQRs), and categorical variables are expressed as numbers (percentages). Continuous variables were analyzed by t-test or the Mann Whitney U-test, and categorical variables were assessed by the chi-square test. Univariate and multivariate linear regression analyses were then performed, while controlling for age and BMI, to further evaluate the relationship between RBP4, and hs-CRP concentrations and AMH levels. The statistically significant setting was *P* < 0.05. All data were processed and analyzed using SPSS 25.0 software.

## Results

### Subject characteristics and laboratory measurements

Demographic, and related variables of IVF treatment and laboratory parameters for all participants are listed in Table 1 and Table 2. There were no differences regarding BMI, estradiol or seasons of blood sample collected. Although, the general age ranges of these two groups were similar, the age of the patients in the DOR group was higher than that of the patients in the NOR group (36.00 ± 6.00 years vs 31.25 ± 4.16 years, *P* < 0.001). As expected, the DOR group had a higher day-3 FSH level (13.02 ± 4.39 mIU/ml vs 8.48 ± 2.65 mIU/ml, *P* < 0.001), lower AMH level (0.61 ± 0.33 ng/ml vs 2.87 ± 1.02 ng/ml, *P* < 0.001), and lower total antral follicle count (6.83 ± 4.03 vs 13.00 ± 8.00, *P* < 0.001) than the NOR group. After IVF treatment, fewer oocytes were retrieved from the DOR group than from the NOR group (3.00 ± 5.00 vs 11.15 ± 5.21, *P* < 0.001). In addition, compared with that of the NOR group, the RBP4 levels (20,648.36 ± 5475.16 ng/ml vs 23,986.48 ± 5995.64 ng/ml, *P* < 0.05) were decreased, and the hs-CRP levels (695.08 ± 1090.19 ng/ml vs 364.32 ± 786.29 ng/ml, *P* < 0.01) were increased.

**Table 1:** Demographic and IVF related characteristics of the patients

Variables	DOR (***n*** = 24)	NOR (***n*** = 48)	***P***
Age (years)	36.00 ± 6.00	31.25 ± 4.16	0.000*
BMI (kg/m^2^)	22.36 ± 2.85	21.20 ± 2.59	0.088
Infertility duration (years)	3.00 ± 5.00	4.00 ± 5.00	0.129
AMH (ng/ml)	0.61 ± 0.33	2.87 ± 1.02	0.000*
Day-3 FSH (mIU/ml)	13.02 ± 4.39	8.48 ± 2.65	0.000*
Estradiol (pg/ml)	45.00 ± 23.39	44.19 ± 13.28	0.820
Season of blood sample, n (%)			
Autumn	13 (54.17%)	30 (62.50%)	
Summer	11 (45.83%)	18 (37.50%)	0.497
Total antral follicle count	6.83 ± 4.03	13.00 ± 8.00	0.000*
Number of oocytes retrieved	3.00 ± 5.00	11.15 ± 5.21	0.000*

Continuous variables are presented as means ± SDs or medians ± interquartile ranges (IQRs). Categorical variables are expressed as numbers (percentages). *DOR* Diminished ovarian reserve, *NOR* Normal ovarian reserve. *P<0.05 indicates statistical significance

**Table 2:** Biochemical parameters of patients

Variables	DOR (***n*** = 24)	NOR (***n*** = 48)	***P***
RBP4 (ng/ml)	20,648.36 ± 5475.16	23,986.48 ± 5995.64	0.025*
hs-CRP (ng/ml)	695.08 ± 1090.19	364.32 ± 786.29	0.012*

Data were presented as means ± SDs or medians ± interquartile ranges (IQRs). *DOR* Diminished ovarian reserve, *NOR* Normal ovarian reserve, *RBP4* Retinol-binding protein 4, *hs-CRP* High sensitivity C-reactive protein. *P<0.05 indicates statistical significance

### Univariable and multivariable regression analyses

**Fig. 2:**
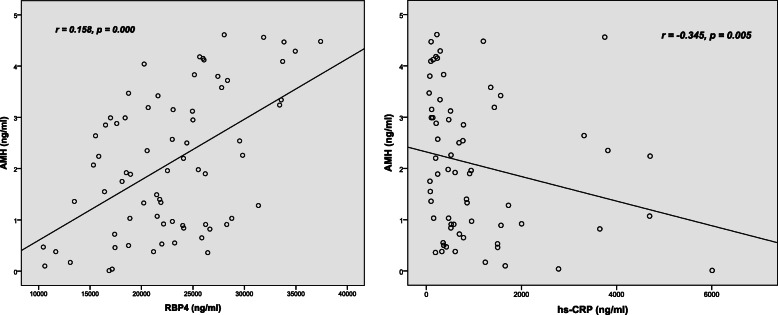
Correlation between serum retinol-binding protein (RBP4), high sensitivity C-reactive protein (hs-CRP) and anti-Mullerian hormone (AMH) levels. RBP4 and AMH are jointly normally distributed data, while hs-CRP is nonnormally distributed continuous data. The Pearson correlation coefficient is used for RBP4 and AMH in all subjects (*n* = 72). The Spearman rank correlation coefficient is used for hs-CRP and AMH in all subjects (*n* = 72). RBP4: Retinol-binding protein 4; hs-CRP: High sensitivity C-reactive protein. *P<0.05 indicates statistical significance

Univariable line regression analysis found significant correlations between RBP4 and AMH (Pearson *r* = 0.518, *p* = 0.000) and between hs-CRP and AMH (Spearman’s *r* = − 0.345, *p* = 0.005) (Fig. 2). After adjustments were made for potential confounders (age and BMI), the results of multiple linear regression analysis showed no association between serum levels of hs-CRP and AMH levels (β = − 0.145, *p* = 0.153); however, a positive relationship between RBP4 and AMH still existed (β = 0.450, *p* = 0.000) (Table 3).

**Table 3:** Regression analysis between covariates and AMH levels

Variables	Unstandardized coefficients (B)	SE	Standardized coefficients (β)	*t* value	*P* value
Age	−0.096	0.031	−0.307	−3.068	0.003*
BMI	−0.058	0.053	−0.111	−1.099	0.276
RBP4	0.000	0.000	0.450	4.409	0.000*
hs-CRP	0.000	0.000	−0.145	−1.1446	0.153
Constant	4.274	1.699		2.516	0.015*

Data were analyzed by multiple liner regression. *BMI* Body mass index, *RBP4* Retinol-binding protein 4, *hs-CRP* High-sensitivity C-reactive protein, *SE* Standard error. The adjusted R^2^: 0.366. *P<0.05 indicates statistical significance

## Discussion

In this cross-sectional study, the concentrations of RBP4 and hs-CRP in the plasma of DOR patients were explored for the first time. We found lower RBP4 levels and higher hs-CRP levels in DOR subjects and a positive association between RBP4 and AMH. After adjustments were made for age and BMI as covariates, this relationship still existed. hs-CRP was not associated with AMH; therefore, RBP4 seems to be associated with ovarian reserve.

Like vitamin E, vitamin A (retinol), is an antioxidant [8]. Because retinol is easily decomposed when exposed to light, coupled with the limitations of detection technology, RBP4 is considered to be a good indicator of serum retinol levels in some cases [18]. RBP4 has been recognized as an adipokine and as one of the oxidative stress markers involved in the pathogenesis of obesity-related metabolic diseases [23]. However, most of these studies were focused on adipose tissues, in which the effects of RBP4 were believed to be attributed to apo-RBP4, a form of RBP4 that does not bind retinol [24–26]. Some studies have shown that the antioxidative effects of RBP4, also known as RBP, may be related to holo-RBP4, which refers to RBP4 combined with retinol [27]. Retinol/RBP (holo-RBP4) was the primary form of retinol in the blood, and its proportion reaches 99% under fasting condition [28]. Previous studies have shown that the levels of serum RBP in retinol-depleted rats was extremely low [29], after administration of retinol, holo-RBP was secreted immediately into the serum [30]. Under several pathophysiological conditions, decreased serum RBP4 levels were related to impaired synthesis and/or secretion of RBP4. This was common in several hepatopathies [31], infection [32] or vitamin A deficiency [33]. In our study, we preliminary ruled out women with any history of liver-related diseases and infection disease, and collected fasting blood, so as to better reflect retinol concentration in the blood.

Due to limitations of commercial Elisa kits, it is impossible to distinguish the above two kinds of RBP4 and evaluated the total RBP4 levels. However, in vitro studies have revealed that apo-RBP4 induced inflammatory cascades [34]. Clinical studies have also found elevated apo-RBP4 in obese subjects and type 2 diabetes [23]. One explanation for this phenomenon may be the lack of retinol in fat, which was insufficient to bind RBP, then apo-RBP was released from the adipose into the blood, thereby diluting serum holo-RBP concentration. Therefore, increased RBP4 levels in the blood of patients with obesity and metabolic diseases does not mean increased levels of retinol. Indeed, the results of Erikstrup C et al.’s study on patients with type 2 diabetes further confirmed this view [24]. They found lower levels of retinol in the blood of such patients, Thus, the concentration of apo-RBP4 was strongly associated with BMI. In our study, we set an even stricter boundary and exclude all people who were obese, and 94.5% of the participants had a BMI of 25 kg/m^2^ or less in our study. Additionally, those at risk of any metabolic diseases were excluded. Therefore, to a certain extent, we controlled for the factors that led to changes in apo-RBP4 levels so that RBP4 levels could better predict blood retinol levels. This view was verified in another study. Hermsdorff et al. [35] found that vitamin A intake and RBP4 levels were positively correlated in Hispanic women who were healthy and not obese (BMI<30 kg/m^2^).

Ovarian dysfunction caused by oxidative stress has been reported to be associated with DOR. Long-term low-dose use of antioxidants containing vitamin A can protect the ovary from ROS induced by oxidative stress [5, 6, 9]. According to prior studies, the accumulation of ROS in granulosa cells caused ovary response poor to FSH in older women through downregulation of follicle-stimulating hormone (FSHR) expression and dysregulation of the FSHR signal transduction pathway [36, 37]. The physiological function of granulosa cells and follicular development is inseparable from the communication between FSH and its receptor. Any changes in FSHR, including reduced contact between FSHR and its ligand and reduced signaling after contacting, may result in decreased ovarian reserve [38]. To the best of our knowledge, fewer studies have evaluated the effect of abnormal retinol alone on ovarian reserve. However, these studies indirectly evaluated the correlation between RBP4 and FSHR. Overexpression of RBP4 can upregulate the expressions of FSHR in granulosa cells [39]. This fact was further supported by Rebeca et al. [40]. As ovarian reserve decreases, FSHR expression in the ovary decreases. Similar to this study, Zeinab et al. [41] also showed a downward trend in FSHR transcript expression in DOR patients. However, in this study, the difference was not significant, and they attributed it mainly to the limited sample size. Contrary to the above findings, Hattori, M.et al. [42] found that retinoic acid (RA), the main active form of retinol in vivo, inhibited FSHR expression, thereby preventing immature granulosa cells from further developing into mature cells. However, the contradictory findings on the effects of retinol and its binding protein and derivates on granulosa cells during follicle development may be because the studies involved different species, examined different groups of subjects, were conducted in diverse geographical areas, had variability in sample size and used different experimental methods. These studies collectively suggested that retinol may regulate ovarian reserve. Hence, it is reasonable to speculate that abnormal RBP4 levels may be associated with DOR. According to our study, the serum RBP4 levels of DOR patients were lower than those of NOR patients, and RBP4 levels were positively correlated with AMH. This association has not been studied or proved before. This association may just be a link, rather than a necessary causal relationship. Nevertheless, it provides a reference basis for our future research.

Oxidative stress induces an abnormal immune response, together with an increase in inflammatory markers. Among them, CRP is a classic marker of chronic inflammation [43] and has been shown to be significantly related to DOR. Two cross-sectional studies found increased CRP in young women (under the age of 35 years) with DOR [19, 20]. Our observation that hs-CRP levels are higher in DOR patients is in line with previous studies. However, after we adjusted for covariates, hs-CRP was not associated with AMH. In previous studies that addressed the relationship between hs-CRP and AMH, there was either a negative correlation [19, 20] or no correlation [44], so the relationship between them still needs to be explored. In a large cross-sectional study, scientists analyzed a group of women aged 21–64 years and suggested negatively correlations between AMH levels and BMI or, fasting glucose. However, we did not observe the same relationship between AMH and BMI. It is worth noting that the BMI levels of the people in our study were all below 28, and 94.5% of them had a BMI of 25 kg/m^2^ or less, this criterion was more stringent than the standards used in previous studies. Our study included only women with normal serum levels of lipids and fasting glucose and women with no family history of cardiovascular diseases because abnormalities in these biochemical parameters and a family history of cardiovascular diseases may impact the plasma levels of CRP and AMH.

To the best of our knowledge, this study is the first to explore the potential connection between the ovarian reserve marker AMH and RBP4, hs-CRP. This research has the following advantages: First, we assessed a group of patients within a normal BMI range and who were under the age of 40 years and had no metabolic risk factors, to eliminate the additional effects of these factors on AMH and biochemical indicators in this experiment. Second, all of our blood samples were taken before IVF started, considering that different doses and kinds of ovulation drugs may have unknown impacts on inflammation and oxidative stress, thus minimizing the potential measurement bias caused by IVF treatment and providing the possibility for an accurate assessment of ovarian function.

Our research has limitations. First, the cross-sectional character of the study that did not able to follow-up of participants at a later stage. Second, we did not perform a direct analysis of retinol but did use RBP4 instead. This was because RBP4 is more stable than retinol and because RBP4 detection kits are less expensive and more easily obtained than retinol detection kits. Third, we did not go on to assess this association in the follicular fluid of the patients due to the small sample sizes of the follicular fluid we collected. Currently, we are still enrolling patients who meet the criteria and collecting both blood and follicular fluid for further research. Fourth, the small number and scope of the research population is mainly related to our strict inclusion and exclusion criteria. It is necessary to further expand the research to include a broader patient group. Nevertheless, our results may provide insight into the potential mechanisms that regulate ovarian reserve.

## Conclusions

In conclusion, decreased serum RBP4 levels and increased serum hs-CRP were first observed in DOR patients in our study, and RBP4 was positively correlated with the ovarian reserve marker AMH. This underlines the fact that oxidative stress plays a role in DOR, and vitamin A may be involved in the recovery of ovarian reserve in DOR patients. However, this association may mainly occur in nonobese women, and whether this phenomenon also occurs in obese DOR patients remains to be determined. Further research should be done in a larger and more diverse population.

## Data Availability

All data generated or analyzed during this study are included in this published article and are available from the corresponding author on reasonable request.

## Abbreviations

DOR: Diminished ovarian reserve
NOR: Normal ovarian reserve
RBP4: Retinol-binding protein 4
hs-CRP: High sensitivity C-reactive protein
AMH: Antimullerian hormone
IVF: In vitro fertilization
ROS: Reactive oxygen species
BMI: Body max index
FSH: Follicle-stimulating hormone
FSHR: Follicle-stimulating hormone receptor
RA: Retinoic acid
